# Oral absorption and drug interaction kinetics of moxifloxacin in an animal model of weightlessness

**DOI:** 10.1038/s41598-021-82044-3

**Published:** 2021-01-28

**Authors:** Dong Liang, Jing Ma, Bo Wei

**Affiliations:** 1 Department of Pharmaceutical Sciences, College of Pharmacy and Health Sciences, Texas Southern University, 3100 Cleburne Street, Houston, TX 77004 USA; 2 Department of Palliative, Rehabilitation and Integrative Medicine, The University of Texas MD Anderson Cancer Center, Houston, 77030 TX USA

**Keywords:** Pharmacokinetics, Translational research

## Abstract

To investigate the effect of simulated weightlessness on the pharmacokinetics of orally administered moxifloxacin and the antacid Maalox or the antidiarrheal Pepto-Bismol using a tail-suspended (TS) rat model of microgravity. Fasted control and TS, jugular-vein-cannulated, male Sprague-Dawley rats received either a single 5 mg/kg intravenous dose or a single 10 mg/kg oral dose of moxifloxacin alone or with a 0.625 mL/kg oral dose of Maalox or a 1.43 mL/kg oral dose of Pepto-Bismol. Plasma concentrations of moxifloxacin were measured by HPLC. Pharmacokinetic data were analyzed using WinNonlin. Simulated weightlessness had no effect on moxifloxacin disposition after intravenous administration but significantly decreased the extent of moxifloxacin oral absorption. The coadministration of moxifloxacin with Maalox to either control or TS rats caused significant reductions in the rate and extent of moxifloxacin absorption. In contrast, the coadministration of moxifloxacin with Pepto-Bismol to TS rats had no significant effect on either the rate or the extent of moxifloxacin absorption. These interactions showed dose staggering when oral administrations of Pepto-Bismol and moxifloxacin were separated by 60 min in control rats but not in TS rats. Dose staggering was more apparent after the coadministration of Maalox and moxifloxacin in TS rats.

## Introduction

Medications are commonly administered during spaceflight^1^. In the past, most medications were used to manage the relatively benign, non-life-threatening problems associated with astronaut adjustment to the space environment during relatively short missions^2,3^. These conditions include space motion sickness, headache, back pain, sleeplessness, nasal congestion and constipation. However, with the development of extensive space missions to the moon and Mars and longer stays on international space stations, astronauts will have an increased opportunity to acquire acute illnesses and infections that are frequently seen on Earth due to impaired immune function and enhanced bacterial growth and expression of virulence factors breathing air in a confined space for extended periods of time^3–5^. Diarrhea caused by an overgrowth of bacteria in the gastrointestinal system and respiratory complications from breathing confined air are the most likely infections that might occur during a mission. Fluoroquinolone antibiotics are a good therapeutic choice in both likely abnormalities because of their strong activity against common gastrointestinal and respiratory pathogens. The bacterial causes of such infections include gram-negative species such as *Pseudomonas aeruginosa* and various *Enterobacteriaceae* and gram-positive species such as *Staphylococcus aureus*, *Enterococci* and *Streptococcus pneumoniae*. All of these species have either developed or acquired antimicrobial resistance to a wide array of standard agents, such as β-lactam antibiotics and cephalosporins. Fluoroquinolones were developed to address specific concerns. Ciprofloxacin was developed as a new weapon against multiresistant gram-negative species, while newer quinolones, including moxifloxacin, were developed to tackle pneumococcal problems and are particularly effective against *S. pneumoniae*.

It is known that weightlessness causes physiological and biomedical changes. Weightlessness could also alter the pharmacokinetics and pharmacodynamics of drugs and thus could impact efficacy and/or toxicity^6,7^. Early exposure to microgravity can cause cephalic body fluid redistribution due to a lack of a hydrostatic pressure gradient. Long-term exposure causes the body to acclimatize to a weightlessness environment, which can lead to deleterious changes such as cardiovascular deconditioning^8^, bone decalcification^9,10^, and a deterioration of weight-bearing muscles^11^. Changes in intrinsic clearance, drug binding to plasma tissue proteins, blood flow and bioavailability can alter drug therapeutic efficacy and toxicity^7,12^. Evidence of possible hepatic metabolic depression due to microgravity exposure^13^ and sex-dependent variations can also potentially contribute to significant alterations in drug pharmacokinetics^14^. The need to understand drug kinetics and pharmacological activities in humans exposed to microgravity increases as technological advancements allow the development of longer, more complex space missions.

Alterations in the rate and/or extent of drug absorption (bioavailability) can significantly affect the clinical efficacy and/or toxicity of drugs. It is well known that in the absence of weightlessness, the bioavailability of orally administered drugs can be appreciably altered by changes in the dissolution rate of the drug, intestinal microflora, intraluminal enzymes, epithelial enzymes, GI membrane permeability, gastric emptying rate, intestinal transit time, and first-pass gut wall and hepatic metabolism^15–17^. The gastrointestinal absorption characteristics of a drug (drug 1) can also be affected by the oral administration of a second drug (drug 2) that interacts with drug 1. However, there is a paucity of information in the literature on the effect of weightlessness on the bioavailability of a drug, and no studies exist in the literature on the effect of microgravity on the absorption kinetics of a drug that physiochemically interacts with a coadministered second drug. Fluoroquinolone antibiotic treatment for respiratory infections is often accompanied by over-the-counter use of medications for concurrent gastrointestinal disorders. Maalox or Pepto-Bismol are the most commonly used antacids and antidiarrheals. In a bioavailability study of healthy volunteers, the coadministration of a single 400 mg dose of moxifloxacin with 10 mL of Maalox 70 resulted in an approximately 60% decrease in the AUC and C_max_. When the antacid was given 4 h before or 2 h after fluoroquinolone, the AUC values were moderately reduced (by < 27%) compared with when moxifloxacin was given alone, and the C_max_ values were almost unchanged^18^. Mass spectrometry assays also identified the formation of a fluoroquinolone-aluminum complex of moxifloxacin with AI^3+^, one of the major ions in Maalox^19^. Metal ions in Maalox (Mg^2+^ and Al^3+^) and Pepto-Bismol (Bi^3+^) could form various types of complexes with moxifloxacin and thus impact its absorption in the GI tract.

**Figure 1:**
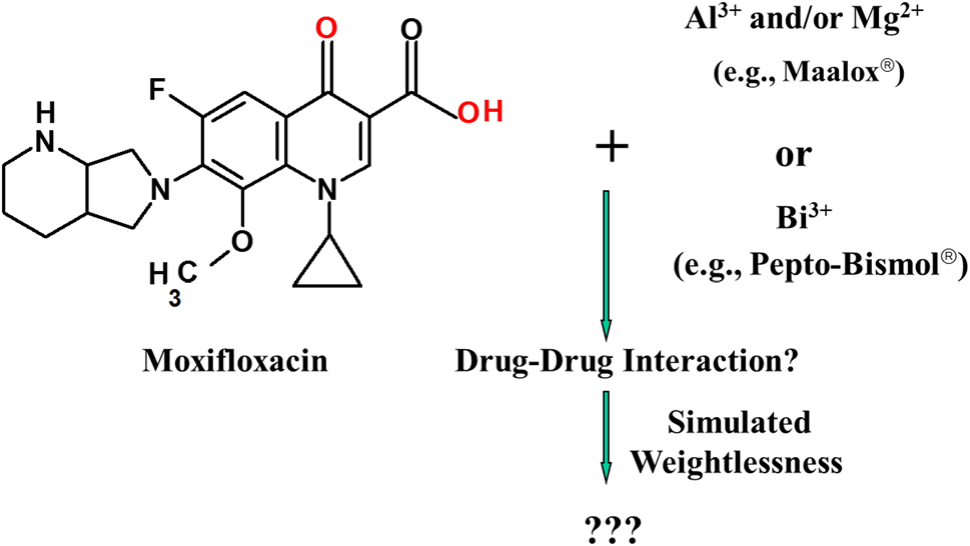
Drug-drug interactions between moxifloxacin and Maalox or Pepto-Bismol.

The tail-suspended rat model is the most accepted and widely used animal model to simulate weightlessness^20^. Rats are suspended by their tail at an angle that produces an approximately 45° head-down tilt, which effectively mimics the physiological changes that occur during actual spaceflights, including bone and muscle atrophy, head and neck edema, increased urine output, dehydration, and urine electrolyte changes. This study is designed to investigate the effects of changes that could occur during spaceflight on the therapeutic effectiveness and adverse drug interactions of moxifloxacin^21,22^ and the over-the-counter medications Maalox or Pepto-Bismol (Fig. 1) using rat tail suspension as a microgravity simulation animal model.

## Results

### Pharmacokinetics of moxifloxacin

The 3-day tail-suspended rat model (TS-3D) had no statistically significant effect (*P* > 0.05) on the disposition (distribution and elimination of intravenously administered moxifloxacin) pharmacokinetic parameters compared to control treatment (Table 1). Following oral administration, however, TS-3D rats had a significantly decreased AUC (46.7% reduction) compared to that of control rats.

**Table 1:** Comparison of mean (SD) pharmacokinetic parameters of moxifloxacin after IV administration of a 5 mg/kg dose or an oral administration of a 10 mg/kg dose to control or tail-suspended rats.

Parameter	Control rats	Tail-suspended rats	Unpaired *t*-test
**Intravenous administration**			
N	8	7	
AUC (mg h/L)	2.14 (0.25)	1.81 (0.54)	NS^a^
T_1/2_ (h)	1.79 (0.34)	1.45 (0.3)	NS
Vss (L/kg)	4.70 (0.43)	4.44 (1.11)	NS
CL (L/kg h)	2.37 (0.29)	3.10 (1.4)	NS
MRT (h)	2.01 (0.28)	1.51 (0.26)	*P* < 0.01
**Oral administration**			
N	10	7	
Cmax (mg/L)	1.65 (0.71)	1.61 (1.4)	NS^a^
AUC (mg h/L)	5.82 (1.6)	3.10 (0.89)	*P* < 0.01
Tmax (h)	0.65 (0.27)	0.40 (0.17)	NS
T_1/2_ (h)	2.24 (0.74)	1.60 (0.3)	*P* < 0.05
CL/F (L/kg h)	1.85 (0.56)	3.43 (0.87)	*P* < 0.01
MRT (h)	3.32 (1.0)	2.30 (0.66)	*P* < 0.05

^a^Not statistically significant.

### Pharmacokinetics of moxifloxacin with bismuth subsalicylate

**Figure 2:**
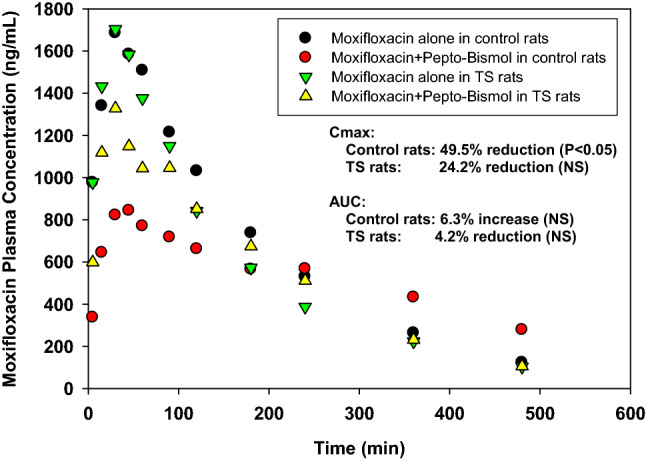
Mean plasma concentration versus time profiles for moxifloxacin after oral administration of a 10 mg/kg dose alone or with Pepto-Bismol either under ground control or simulated weightlessness.

Pharmacokinetic interactions between moxifloxacin and concomitantly administered bismuth subsalicylate under ground control and simulated weightlessness are shown in Fig. 2 and Table 2. In control rats, compared with the administration of moxifloxacin alone, concomitant administration of moxifloxacin 5 min after Pepto-Bismol administration statistically significantly reduced the C_max_ by 49.5% (from 1.73 ± 0.14 mg/L to 0.873 ± 0.15 mg/L) and statistically significantly increased the T½ by 121% (from 1.92 ± 0.26 h to 4.25 ± 0.97 h). This T½ was appreciably longer (twofold; *P* < 0.001) than that observed after IV administration of moxifloxacin alone to control rats (T½ = 1.79 h), indicating that bismuth subsalicylate decreases the rate of absorption of moxifloxacin to such an extent that flip-flop kinetics become evident. MRT was also significantly increased by 122% (from 2.92 ± 0.39 h to 6.48 ± 1.3 h) when moxifloxacin was concomitantly administered with Pepto-Bismol to the control rats. The coadministration of Pepto-Bismol to control rats had no significant effect on the AUC (reflective of the extent of drug absorption or the bioavailability of the drug; AUC = 6.08 ± 1.6 mg·h/L in the presence and 5.72 ± 1.2 mg· h/L in the absence of Pepto-Bismol), the T_max_ (T_max_ = 0.71 ± 0.23 h in the presence and 0.66 ± 0.23 h in the absence of Pepto-Bismol) or the apparent oral clearance of moxifloxacin (CL/F = 1.75 ± 0.46 L/kg· h in the presence and 1.83 ± 0.45 L/kg· h in the absence of Pepto-Bismol).

**Table 2:** Comparison of mean (SD) pharmacokinetic parameters of moxifloxacin after the administration of a 10 mg/kg oral dose alone or with a 1.43 mL/kg oral dose of pepto-bismol suspension^a^ to control and tail-suspended rats.

Parameter	Moxifloxacin alone (M)	Moxifloxacin + pepto-bismol (MPB)	Unpaired *t*-test	Difference^b^
**Control rats**				
N	8	7		
Cmax (mg/L)	1.73 (0.14)	0.873 (0.15)	*P* < 0.002	49.5%
Tmax (h)	0.66 (0.23)	0.71 (0.23)	NS^c^	–
T_1/2_ (h)	1.92 (0.26)	4.25 (0.97)	*P* < 0.002	121%
AUC (mg h/L)	5.72 (1.2)	6.08 (1.6)	NS	–
CL/F (L/kg h)	1.83 (0.45)	1.75 (0.46)	NS	–
MRT (h)	2.92 (0.39)	6.48 (1.3)	*P* < 0.002	122%
**Tail-suspended rats**				
N	6	7		
Cmax (mg/L)	1.61 (0.43)	1.33 (0.35)	NS	–
Tmax (h)	0.54 (0.19)	0.50 (–)	NS	–
T_1/2_ (h)	1.87 (0.39)	1.73 (0.19)	NS	–
AUC (mg h/L)	4.07 (1.6)	4.76 (1.5)	NS	–
CL/F (L/kg h)	2.86 (1.3)	2.31 (0.76)	NS	–
MRT (h)	2.45 (0.44)	3.00 (0.27)	*P* < 0.02	22.4%

^a^Administered 5 min before moxifloxacin and containing 50 mg/kg bismuth subsalicylate.

^b^Absolute value of [(M-MPB)/M] * 100.

When compared with the mean pharmacokinetic parameters observed in control rats, there was no statistically significant effect (*P* > 0.05) of concomitant oral administration of Pepto-Bismol on either the rate or the extent of moxifloxacin absorption in TS-3D rats (absence vs. presence of Pepto-Bismol).

### Pharmacokinetics of moxifloxacin with aluminum/magnesium hydroxide

**Figure 3:**
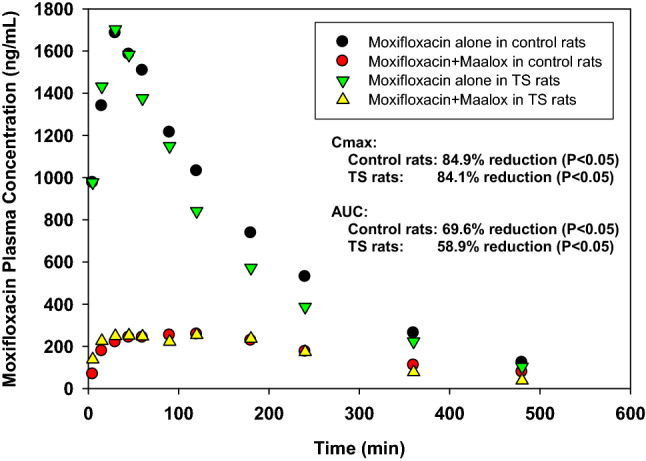
Mean plasma concentration versus time profiles for moxifloxacin after oral administration of a 10 mg/kg dose alone or with Maalox either under ground control or simulated weightlessness.

Pharmacokinetic interactions between moxifloxacin and concomitantly administered aluminum/magnesium hydroxide under control and simulated weightlessness are shown in Fig. 3 and Table 3. In control rats, concomitant oral administration of moxifloxacin with Maalox caused pronounced and statistically significant reductions in both the rate and the extent of moxifloxacin absorption compared with the administration of moxifloxacin alone. The C_max_ was reduced by 84.9% (from 1.73 ± 0.14 mg/L in the absence to 0.261 ± 0.04 mg/L in the presence of Maalox), the AUC was reduced by 69.6% (from 5.72 ± 1.2 mg· h/L in the absence to 1.74 ± 0.43 mg· h/L in the presence of Maalox), the T_max_ was increased by 115% (from 0.66 ± 0.23 h in the absence to 1.42 ± 0.56 h in the presence of Maalox), the T½ was increased by 68.2% (from 1.92 ± 0.26 h in the absence to 3.23 ± 0.55 h in the presence of Maalox), the CL/F was increased by 231% (from 1.83 ± 0.45 L/kg· h in the absence to 6.05 ± 1.4 L/kg· h in the presence of Maalox), and the MRT was significantly elevated by 79.1% (from 2.92 ± 0.39 h in the absence to 5.23 ± 0.86 h in the presence of Maalox). Similar to the flip-flop kinetics observed when moxifloxacin was coadministered with Pepto-Bismol, the T½ of moxifloxacin coadministered with Maalox (T½ = 3.23 h) was appreciably longer (*P* < 0.05) than that observed after IV administration of moxifloxacin alone to control rats (T½ = 1.79 h).

**Table 3:** Comparison of mean (SD) pharmacokinetic parameters of moxifloxacin after the administration of a 10 mg/kg oral dose alone or with a 0.625 mL/kg oral dose of maalox suspension^a^ to control and tail-suspended rats.

Parameter	Moxifloxacin alone	Moxifloxacin + maalox	Unpaired *t*-test	Difference^b^
**Control rats**				
N	8	6		
Cmax (mg/L)	1.73 (0.14)	0.261 (0.04)	*P* < 0.00001	84.9%
Tmax (h)	0.66 (0.23)	1.42 (0.56)	*P* < 0.005	115%
T_1/2_ (h)	1.92 (0.26)	3.23 (0.55)	*P* < 0.0001	68.2%
AUC (mg-h/L)	5.72 (1.2)	1.74 (0.43)	*P* < 00001	69.6%
CL/F (L/kg h)	1.83 (0.45)	6.05 (1.4)	*P* < 0.00001	231%
MRT (h)	2.92 (0.39)	5.23 (0.86)	*P* < 0.00002	79.1%
**Tail-suspended rats**				
N	8	7		
Cmax (mg/L)	1.75 (0.43)	0.279 (0.08)	*P* < 0.00001	84.1%
Tmax (h)	0.69 (0.59)	1.61 (1.2)	NS (* P* > 0.05)	133%
T_1/2_ (h)	2.13 (0.57)	2.20 (0.33)	NS (* P* > 0.05)	3.29%
AUC (mg-h/L)	3.48 (1.3)	1.43 (0.44)	*P* < 0.002	58.9%
CL/F (L/kg-h)	3.29 (1.4)	7.52 (2.0)	*P* < 0.0005	129%
MRT (h)	2.78 (0.49)	3.81 (0.34)	*P* < 0.0005	37.1%

^a^Administered 5 min before moxifloxacin and containing 50 mg/kg Al(OH)_3_ and 50 mg/kg Mg(OH)_2_.

^b^Absolute value of [(M-MMX)/M] * 100.

Both the rate and the extent of moxifloxacin absorption were also statistically significantly lower in the TS-3D rats when moxifloxacin was orally coadministered with Maalox. The C_max_ was reduced by 77% (from 1.21 ± 0.21 mg/L in the absence to 0.279 ± 0.08 mg/L in the presence of Maalox), the AUC was reduced by 58.9% (from 3.48 ± 1.3 mg· h/L in the absence to 1.43 ± 0.44 mg· h/L in the presence of Maalox), the CL/F was increased by 129% (from 3.29 ± 1.4 L/kg·h in the absence to 7.52 ± 2.0 L/kg·h in the presence of Maalox), and the MRT was increased by 31.7% (from 2.78 ± 0491 h in the absence to 3.81 ± 0.34 h in the presence of Maalox). The mean T_max_ and T½ values were not significantly affected by concomitantly administered Maalox.

For both the control and TS-3D rats, the impairment noted in the rate and extent of GI absorption can be attributed to the formation of nonabsorbable chelate complexes between Mg^2+^ and Al^3+^ ions in Maalox and moxifloxacin molecules.

### Effect of time interval between moxifloxacin and pepto-bismol /maalox administration on absorption kinetics

Pharmacokinetic parameters for moxifloxacin following oral administration to control rats of moxifloxacin alone and with Pepto-Bismol dosed at − 5 min and − 60 min are presented in Table 4. The coadministration of Pepto-Bismol (the − 5-min group) caused a significant reduction in the magnitude of the C_max_ for moxifloxacin but had no significant effect on the magnitude of the AUC for moxifloxacin. The terminal phase half-life for moxifloxacin in the control rats was significantly increased by Pepto-Bismol. This drug-drug interaction and the resultant reduction in the rate of antibiotic absorption still occurred but to a statistically significant reduced degree when oral administrations of Pepto-Bismol and moxifloxacin were separated by 60 min.

**Table 4:** Comparison of mean (± SD) pharmacokinetic parameters of moxifloxacin after a 10 mg/kg oral dose alone or with a 1.43 mL/kg oral dose of pepto-bismol suspension^a^ administered at − 5 min or − 60 min to control male rats under fasting conditions.

Parameter	Moxifloxacin alone (M)	Moxifloxacin + pepto-bismol (MP)	
**Control rats**			
Administration of pepto-bismol		5 min before moxifloxacin	60 min before moxifloxacin
N	8	7	8
Cmax (mg/L)	1.729 (0.144)	0.873 (0.153)^b^	1.305 (0.317)^c,d^
Tmax (h)	0.66 (0.23)	0.71 (0.22)	0.54 (0.35)
T_1/2_ (h)	1.92 (0.26)	4.26 (0.42)^b^	2.11 (0.42)^d^
AUC (mg h/L)	5.72 (1.2)	6.08 (1.7)	4.64 (1.4)
CL/F (L/kg h)	1.83 (0.45)	1.75 (0.46)	2.32 (0.65)
MRT (h)	2.92 (0.39)	6.48 (1.3)^b^	3.33 (0.72)^d^
**Tail-suspended rats**			
Administration of pepto-bismol		5 min before moxifloxacin	60 min before moxifloxacin
N	9	7	4
Cmax (mg/L)	1.751 (0.428)	1.328 (0.354)	1.663 (0.364)
Tmax (h)	0.53 (0.15)	0.50 (0.00)	0.38 (0.14)^b^
T_1/2_ (h)	1.97 (0.35)	1.73 (0.19)	2.16 (0.25)^b^
AUC (mg h/L)	4.97 (1.9)	4.76 (1.5)	5.21 (1.5)
CL/F (L/kg h)	2.40 (1.3)	2.31 (0.76)	2.04 (0.57)
MRT (h)	2.69 (0.51)	3.00 (0.27)	3.17 (0.42)

^a^Equivalent to 50 mg/kg bismuth subsalicylate.

^b^Statistically significant (*P* < 0.05, unpaired t-test) compared with parameters obtained from the moxifloxacin alone rat group.

^c^Statistically significant (*P* < 0.05, unpaired t-test) compared with parameters obtained from the moxifloxacin alone rat group.

^d^Statistically significant (*P* < 0.05, unpaired t-test) compared with parameters obtained from the Pepto-Bismol at − 5 min rat group.

The separation of Pepto-Bismol and moxifloxacin oral doses by 60 min significantly lessened the reductive effects of bismuth subsalicylate on the absorption rate of moxifloxacin in control rats compared to the separation of their doses by 5 min. By increasing the time interval between the interacting doses to 60 min, the mean C_max_ value was significantly increased by 50.1% (0.873 ± 0.15 mg/L for the − 5-min group vs. 1.31 ± 0.32 mg/L for the − 60-min group), the mean T½ value was significantly reduced by 50.5% (4.26 ± 0.42 h for the − 5-min group vs. 2.11 ± 0.42 h for the − 60-min group), and the mean MRT value was significantly reduced by 48.6% (6.48 ± 1.3 h for the − 5-min group vs. 3.33 ± 0.72 h for the − 60-min group).

Irrespective of the administration time interval, Pepto-Bismol had no statistically significant effect on either the rate or the extent of moxifloxacin absorption in TS-3D rats. In striking contrast, oral administration to 3-day TS rats of moxifloxacin with Pepto-Bismol dosed at either − 5 min or − 60 min had no significant effect on either the rate of moxifloxacin absorption or the extent of moxifloxacin absorption (Table 4).

The separation of Maalox and moxifloxacin oral doses by 60 min did not significantly lessen the pronounced reductive effects of aluminum and magnesium hydroxides on the rate and extent of absorption of concomitantly administered moxifloxacin to control rats compared to the separation of these doses by 5 min. No statistically significant difference existed between the − 5-min and − 60-min control groups with respect to any absorption parameter except Cmax (Table 5).

**Table 5:** Comparison of mean (± SD) pharmacokinetic parameters of moxifloxacin after a 10 mg/kg oral dose alone or with a 0.625 mL/kg oral dose of maalox suspension^a^ administered at − 5 min or − 60 min to control male rats or tail-suspended (TS) male rats under fasting conditions.

Parameter	Moxifloxacin alone (M)	Moxifloxacin + maalox (MM)	
**Control rats**			
Administration of maalox		5 min before moxifloxacin	60 min before moxifloxacin
N	8	6	7
Cmax (mg/L)	1.729 (0.144)	0.261 (0.037)^b^	0.316.3 (0.041)^c,d^
Tmax (h)	0.66 (0.23)	1.42 (0.56)^b^	1.86 (1.1)^c^
T_1/2_ (h)	1.92 (0.26)	3.23 (0.55)^b^	2.71 (0.44)^c^
AUC (mg h/L)	5.72 (1.2)	1.74 (0.43)^b^	2.10 (0.19)^c^
CL/F (L/kg h)	1.83 (0.45)	6.05 (1.4)^b^	4.80 (0.44)^c^
MRT (h)	2.92 (0.39)	5.23 (0.86)^b^	5.01 (0.48)^c^
**Tail-suspended rats**			
Administration of maalox		5 min before moxifloxacin	60 min before moxifloxacin
N	9	7	4
Cmax (mg/L)	1.751 (0.428)	0.278 (0.082)^b^	0.689 (0.164)^c,d^
Tmax (h)	0.53 (0.15)	1.61 (1.2)	0.43 (0.13)^d^
T_1/2_ (h)	1.97 (0.35)	2.20 (0.33)	2.68 (0.60)^c^
AUC (mg h/L)	4.97 (1.9)	1.43 (0.44)^b^	3.23 (0.22)^c,d^
CL/F (L/kg h)	2.40 (1.3)	7.52 (2.0)^b^	3.10 (0.22)^d^
MRT (h)	2.69 (0.51)	3.81 (0.34)^c^	4.16 (1.1)^c^

^a^Equivalent to 50 mg/kg Al(OH)_3_ and 50 mg/kg Mg(OH)_2_.

^b^Statistically significant (*P* < 0.05, unpaired t-test) compared with parameters obtained from the moxifloxacin alone rat group.

^c^Statistically significant (*P* < 0.05, unpaired t-test) compared with parameters obtained from the moxifloxacin alone rat group.

^d^Statistically significant differences (*P* < 0.05, unpaired t-test) compared with parameters obtained from the maalox-5 min rat group.

When compared with those observed in control rats, the pronounced reductive effects of aluminum and magnesium hydroxides on the rate and extent of absorption of concomitantly administered moxifloxacin were appreciably and significantly lessened by the separation of Maalox and moxifloxacin by 60 min in TS-3D rats. By increasing the time interval between the interactant doses to 60 min, the mean C_max_ value was significantly increased by 147% (0.279 ± 0.082 mg/L for the − 5-min group vs. 0.690 ± 0.16 mg/L for the − 60-min group); the mean T½ value was significantly decreased by 73.3% (1.61 ± 1.2 h for the − 5-min group vs. 0.43 ± 0.13 h for the − 60-min group); the mean AUC value was increased by 126% (1.43 ± 0.44 mg·h/L for the − 5-min group vs. 3.23 ± 0.22 mg·h/L for the − 60-min group); and the mean CL/F value was reduced by 58.8% (7.52 ± 2.0 L/kg·h for the − 5-min group vs. 3.10 ± 0.22 L/kg·h for the − 60-min group) (Table 5).

## Discussion

Limited information is available regarding the pharmacokinetic and pharmacodynamic effects of a drug when administered in space^1,6^. Microgravity exposure causes physiological and biomedical changes in the human body that can significantly alter drug efficacy and toxicity. NASA’s Biomedical Research and Countermeasure Program has given the need to evaluate the pharmacokinetics of antibiotics for use in space missions a high priority. Broad-spectrum antibiotics such as fluoroquinolones have excellent activity against the pathogens that are most likely to be encountered during space missions. Using the tail-suspension model^20^ to simulate microgravity, the absorption and disposition kinetics of moxifloxacin and other fluoroquinolone antibiotics (ciprofloxacin, gatifloxacin and levofloxacin) were found to be unaffected by simulated weightlessness. Our results are consistent with the findings of Schuck et al*.*^23^, who reported that the disposition kinetics, plasma protein binding and tissue penetration of ciprofloxacin were unchanged following 3 days of 6° antiorthostatic bed rest in a human model of simulated weightlessness. It is worth noting that the tail-suspension rat model and the head-down bedrest human model have been generally accepted as ground-based simulated weightlessness models for pharmacokinetic studies^16^.

Following intravenous administration of moxifloxacin to control rats, the pharmacokinetic parameters shown in our study were consistent with published data. For example, our mean systemic clearance and volume of distribution of moxifloxacin were 2.37 L/h-kg and 4.7 L/kg, respectively, as compared to 2.55 L/h-kg and 3.6 L/kg, respectively, reported by Siefert et al*.*^24^ There was no significant effect of simulated weightlessness on pharmacokinetic parameters of intravenously administered moxifloxacin. However, following oral administration, the extent but not the rate of absorption of moxifloxacin was significantly decreased under simulated microgravity. This observed insufficient absorption was most likely due to the increased gastric emptying and intestinal transit time induced by microgravity^15^.

It is known that fluoroquinolone antibiotics interact significantly and extensively with metal ions in the GI tract. The formation of nonabsorbable fluoroquinolone-metal complexes in the GI tract often results in changes in the bioavailability of fluoroquinolone drugs^25^. Our studies on the pharmacokinetics of the interaction between moxifloxacin and Pepto-Bismol have shown that the TS-3D rat model, as a model of simulated weightlessness, can alter these types of drug-metal ion interactions. The rate but not the extent of moxifloxacin absorption was markedly reduced when Pepto-Bismol was given either 5 min or 60 min before oral administration of moxifloxacin to control rats. The formation of a nonabsorbable chelate complex between the Bi^3+^ ions in the Pepto-Bismol and moxifloxacin molecules is the cause of the marked decrease in the absorption rate. The chelate complex of moxifloxacin and Bi^3+^ ions, however, might be weak and reversible in the GI tract and thus result in the observed impact on the rate but not the extent of absorption. For example, commonly used doses of ferrous sulfate, zinc sulfate, aluminum hydroxide and magnesium hydroxide, but not bismuth subsalicylate, reduced the absorption of norfloxacin by 50 to 90% in healthy volunteers^26^. Rambout et al*.*^27^ reported no significant effect of ciprofloxacin bioavailability after coadministration with bismuth subsalicylate in healthy volunteers. In fact, moxifloxacin has been successfully used clinically with bismuth as a quadruple therapy for first-line treatment of *Helicobacter pylori* infection^28^.

In striking contrast, there was no significant effect on either the rate or the extent of moxifloxacin absorption when administered by mouth, either 5 or 60 min, after Pepto-Bismol administration to TS-3D rats. The results in TS-3D rats suggest that simulated weightlessness may have affected the equilibrium between free moxifloxacin and the moxifloxacin-bismuth ion chelate complex. It is also possible that a microgravity-induced alteration in gastrointestinal transit time effectively decreased the proximity of the two interactants to each other.

The interaction observed between moxifloxacin and Maalox was much stronger than the interaction observed between moxifloxacin and Pepto-Bismol. The rate and extent of oral moxifloxacin absorption was markedly impaired when moxifloxacin was given concomitantly with Maalox to both control and TS-3D rats. Maalox also significantly increased the terminal half-lives of moxifloxacin in control rats but not in TS-3D rats. In control rats, even when the doses were separated by 60 min, statistically significant impairment of absorption still occurred. However, the effect on absorption was significantly lessened when Maalox was administered 60 min before oral moxifloxacin in TS-3D rats. The results suggest that a further increase in the time interval between oral doses of Maalox and moxifloxacin can possibly prevent this interaction altogether. Complex formation between aluminum(III) ions (the main interacting ion in Maalox) and fluoroquinolone antibiotics such as moxifloxacin is known^29^. Our study results are consistent with those of a prior report by Stass et al*.*^30^, where concomitant ingestion with sucralfate and/or oral aluminum(III) ion-containing antacids significantly reduced the bioavailability of moxifloxacin in healthy volunteers.

However, the underlying mechanism of this dose-staggering effect is unclear. We speculate that either Mg^2+^ or Al^3+^ ions from the Maalox are absorbed and less available or move along in the GI tract apart from moxifloxacin after dose staggering. Amidon et al*.*^15^ indicated that in space under microgravity, gastric emptying and intestinal transit time are decreased, which will lead to inefficient absorption. This would explain the dose-staggering effect of Maalox and moxifloxacin in our study. After oral administration of magnesium hydroxide, Mg^2+^ concentrations in plasma showed a sustained and continued absorption of up to 12 h with a bioavailability of 15% in healthy volunteers^31^. Mg^2+^ is absorbed via a passive paracellular pathway and an active transcellular pathway that involves TRPM6/7 channel proteins^32^. The absorption of aluminum depends on the nature of the ligands associated with the Al^3+^ ion in the gastrointestinal fluid. The higher aluminum concentration is always in the jejunal fragment because of the influence of pH variation on this fragment^32^. In the case of Pepto-Bismol, Nwokolo et al*.*^33^ showed that despite rapid and substantial absorption of salicylate, there was negligible absorption of bismuth into the bloodstream from standard oral doses of bismuth salicylate.

It is worth noting that fluoroquinolone antibiotics such as moxifloxacin undergo minimal hepatic metabolism. The majority of the drug is excreted unchanged in the urine. Since many drug interactions observed under weightlessness were related to hepatic drug metabolism, our findings are consistent with prior literature reports focusing on hepatic enzymatic activity changes under weightlessness^4,10,34^. On the other hand, the metal ions investigated in this study, i.e., Mg^2+^, Al^3+^, and Bi^3+^, have pharmacological and therapeutic functions^34,35^. However, the effect of weightlessness on the pharmacokinetics of coadministered Maalox or Pepto-Bismol, i.e., Mg^2+^, Al^3+^, and Bi^3+^, is unknown.

Gastrointestinal changes under weightlessness have been attributed to changes in drug absorption^6,15,36^. It is known that drug pharmacokinetics may change under simulated weightlessness due to a change in gastric emptying time^37^ or hepatic drug metabolism^38^. Limited information is available on how gastric emptying and intestinal transit rate and time will impact oral drug-drug interactions and absorption. Roda et al*.*^39^ reported that ^13^C-based breath tests were suitable for monitoring gastrointestinal motility in the 520-d isolation experiment within the MARS-500 project and can be applied in long-term spaceflights. Our study provides some insight into drug-drug interactions that can occur in the gastrointestinal tract during spaceflight that are different from those that have been observed under ground control conditions. Our study showed a lesser degree of reduction in the rate and extent of moxifloxacin absorption when it was coadministered with Maalox or Pepto-Bismol under simulated weightlessness than when administered under ground control. Even though this study was not a human clinical trial, nor did it take place under real spaceflight conditions, our findings provide experimental information for clinicians on potential drug-drug interactions in spaceflight.

The present study has certain limitations. For example, the tail-suspended rat model has not been validated with a human space weightless model to predict the pharmacokinetic behaviors of drugs, although the tail-suspended rat model has been used frequently as a simulated weightlessness model to characterize the pharmacokinetics of drugs^4,10,20,34,38^. The use of only male and relatively young adult rats may limit the scope of discovery in real practice because astronauts are generally not very young, and many of them are females.

## Conclusion

In conclusion, simulated weightlessness had no significant effect on the pharmacokinetic interactions observed from the coadministration of moxifloxacin with Maalox to control rats, but significantly diminished the pharmacokinetic interactions observed from the coadministration of moxifloxacin with Pepto-Bismol to control rats. The simulated weightlessness might contribute to the limited dose-staggering interactions between moxifloxacin and Maalox or Pepto-Bismol due to decreased gastric emptying and intestinal transit times.

## Methods

### Animals

This study was carried out in accordance with the recommendation of the Guidelines for the Care and Use of Laboratory Animals (1996) and was in compliance with the ARRIVE guidelines. All animal experiments were conducted with prior approval from the Texas Southern University Institutional Animal Care and Use Committee (IACUC). Male Sprague-Dawley (SD) rats (250–350 g) were purchased from Harlan Laboratories, Inc. (Indianapolis, IN) and were housed in standard rodent cages (3–4 rats per cage) with a 12-h light/dark cycle at 20 °C and had free access to standard laboratory rodent chow (7012 Harlan Teklad mouse/rat diet from Harlan Laboratories, Inc.) and water. Rats were acclimated to the animal care facility for at least 7 days before the start of each experiment. Only male rats were used in the present study to minimize pharmacokinetic variations due to sex differences. In general, female rats weigh much less than male rats of the same age.

### Materials

Moxifloxacin was provided by Bayer Corporation (West Haven, CT). Acetonitrile and hydrochloride acid were purchased from Fisher Scientific (Pittsburgh, PA). Ketamine hydrochloride, xylazine hydrochloride, and acetopromazine maleate salt were purchased from Sigma Chemical Company. All other chemicals were HPLC grade and purchased from Sigma Chemical Company (St. Louis, MO).

### Procedures

To facilitate the timed withdrawal of multiple blood samples from each animal, the right jugular vein of each control animal was cannulated one day prior to drug administration, while the right jugular vein of each tail-suspended animal was cannulated on the initial day of the tail-suspension period. The cannulas were flushed daily with 0.5 mL sterile heparinized saline (100 units/ml). Under ketamine:acetopromazine:xylazine (50:3.3:3.3 mg/kg ip) anesthesia, silicone elastomer tubing (0.02 × 0.037 in) was inserted into the jugular vein, secured with a silk suture, and exteriorized in the dorsal intrascapular area. The surgical incision was prophylactically treated with nitrofurazone wound powder and closed with surgical staples.

Weightlessness was simulated using the tail-suspended rat model. Rats were isolated in standard tail-suspended rodent cages (provided by Dr. Lane Brunner) and allowed free access to food and water. The tail-suspension procedures were previously described^10,40^.

All animals were fasted for 12 h prior to drug administration but were allowed free access to water at all times. Control (nonweightlessness) rats were treated in a manner similar to the experimental (weightless) rats, with the only difference being that the control rats were not subjected to the tail-suspension procedure. The start of the tail-suspension procedure represented day 0 of the study period. Drug absorption studies were initiated 3 days after the start of the tail-suspension procedure. Subsequent to drug administration, multiple blood samples (0.25 mL) were collected (from the jugular vein cannula) into heparinized tubes immediately before dosing and at 5, 15, 30, 45, 60, 90, 120, 180, 240, 360, and 480 min after moxifloxacin administration. The blood samples were centrifuged, and plasma samples were separated and stored at − 80 °C until HPLC assay.

### Pharmacokinetic studies

To study the effect of microgravity on the systemic clearance and gastrointestinal (GI) absorption kinetics of moxifloxacin using a tail-suspended microgravity rat model, male SD rats were used to assess the disposition kinetics of moxifloxacin (5 mg/kg) after intravenous administration to control (N = 8) and 3-day tail-suspended (TS-3D) rats (N = 7). Another group of male SD rat was used to assess the absorption kinetics of moxifloxacin (10 mg/kg) after oral administration to control (N = 10) and 3-day tail-suspended rats (N = 7). The drug was administered as an aqueous solution. Plasma samples were collected and assayed as described above.

Male SD rats were used to assess quantitatively the effect of bismuth subsalicylate (an antidiarrheal agent and a potential interactant) on the gastrointestinal absorption kinetics of moxifloxacin in a tail-suspended rat model. Moxifloxacin was administered orally (10 mg/kg) alone or with a 1.43 mL/kg oral dose of Pepto-Bismol suspension (equivalent to 50 mg/kg of bismuth subsalicylate) to control and 3-day tail-suspended rats. Plasma samples were collected and assayed as previously described.

- Group #1 (moxifloxacin alone in control rats): N = 8 rats given 10 mg/kg PO moxifloxacin.
- Group #2 (moxifloxacin + Pepto-Bismol in control rats): N = 7 rats given 10 mg/kg PO moxifloxacin and a 1.43 mL/kg dose of Pepto-Bismol.
- Group #3 (moxifloxacin alone in TS-3D rats): N = 6 rats given 10 mg/kg PO moxifloxacin.
- Group #4 (moxifloxacin + Pepto-Bismol in TS-3D rats): N = 7 rats given 10 mg/kg PO of moxifloxacin and a 1.43 mL/kg dose of Pepto-Bismol.

Male SD rats were used to assess quantitatively the effect of the aluminum hydroxide/magnesium hydroxide-containing antacid Maalox (an antacid and potential drug interactant) on the gastrointestinal absorption kinetics of moxifloxacin in a tail-suspended rat model. Moxifloxacin was administered orally (10 mg/kg) alone or with a 0.625 mL/kg oral dose of Maalox suspension. The dose of Maalox suspension was equivalent to 50 mg/kg aluminum hydroxide and 50 mg/kg magnesium hydroxide. Plasma samples were collected and assayed as described in the previous section.

- Group #5 (moxifloxacin alone in control rats): N = 8 rats given 10 mg/kg PO moxifloxacin.
- Group #6 (moxifloxacin + Maalox in control rats): N = 6 rats given 10 mg/kg PO moxifloxacin and a 0.625 mL/kg dose of Maalox.
- Group #7 (moxifloxacin alone in TS rats): N = 8 TS-3D rats given 10 mg/kg PO moxifloxacin.
- Group #8 (moxifloxacin + Maalox in TS rats): N = 7 TS-3D rats given 10 mg/kg PO moxifloxacin and a 0.625 mL/kg dose of Maalox.

To assess quantitatively whether the time interval between the oral administration of moxifloxacin and the oral administration of an aluminum hydroxide/magnesium hydroxide antacid mixture (Maalox) or between the oral administration of moxifloxacin and the oral administration of an antidiarrheal agent bismuth subsalicylate (Pepto-Bismol) can affect the gastrointestinal absorption kinetics of moxifloxacin in a TS microgravity rat model, control and TS male SD rats were orally dosed with moxifloxacin (10 mg/kg) alone or with moxifloxacin with 0.625 mL/kg Maalox suspension (administered at − 5 min or − 60 min) or with moxifloxacin with 1.43 mL/kg Pepto-Bismol suspension (administered at − 5 min or − 60 min). The dose of Maalox suspension was equivalent to 50 mg/kg aluminum hydroxide and 50 mg/kg magnesium hydroxide. The dose of Pepto-Bismol suspension was equivalent to 50 mg/kg bismuth subsalicylate.

- Group #9 (moxifloxacin + Maalox at − 60 min in control rats): N = 7 rats were first given an oral 0.625 mL/kg dose of Maalox, and 60 min later, the rats were given 10 mg/kg PO moxifloxacin.
- Group #10 (moxifloxacin alone in TS-3D rats): N = 9 rats were given 10 mg/kg PO moxifloxacin.
- Group #11 (moxifloxacin + Maalox at − 60 min in TS-3D rats): N = 4 rats were first given an oral 0.625 mL/kg dose of Maalox, and 60 min later, the rats were given 10 mg/kg PO moxifloxacin.
- Group #12 (moxifloxacin + Pepto-Bismol at − 60 min in control rats): N = 8 rats were first given an oral 1.43 mL/kg dose of Pepto-Bismol, and 60 min later, the rats were given 10 mg/kg PO moxifloxacin.
- Group #13 (moxifloxacin + Pepto-Bismol at − 60 min in TS-3D rats): N = 4 rats were first given an oral 1.43 mL/kg dose of Pepto-Bismol, and 60 min later, the rats were given 10 mg/kg PO moxifloxacin.

### HPLC assay for moxifloxacin concentrations in rat plasma

Moxifloxacin was extracted from plasma by mixing 0.1 mL of plasma with 0.1 mL of a ciprofloxacin (as an internal standard)-containing acetonitrile solution to precipitate plasma proteins. After centrifugation, the pH of an aliquot of the supernatant was adjusted to pH 4.0 with assay buffer prior to the injection of the aliquot into the HPLC column. Moxifloxacin and the internal standard were separated on a reverse-phase Xterra C_18_ column (4.6 × 150 mm, 3.5 µm, Waters Corporation, Milford, MA) preceded by an Xterra C_18_ guard column. Chromatograms were developed on an HPLC (model 515 pump) equipped with a 717 plus autosampler, a model 474 fluorescence detector, and a Millennium data system. The mobile phase consisted of 45% acetonitrile in an aqueous solution containing 10 mM sodium dodecyl sulfate, 10 mM tetraethylammonium acetate, and 25 mM citric acid (adjusted to pH 3.50). The flow rate was 1.0 ml/min, and the effluent was monitored at an excitation wavelength of 296 nm and an emission wavelength of 504 nm. Under these conditions, the retention times for moxifloxacin and the internal standard were 5.7 and 8.2 min, respectively. The assay was linear for moxifloxacin concentrations in the range of 12.5–900 ng/mL.

### Data interpretation

#### Pharmacokinetic parameters

Noncompartmental pharmacokinetic parameters [biological half-life (T_½_), plasma clearance (CL), volume of distribution at steady-state (V_ss_), total area under the plasma concentration–time curve (AUC), and mean residence time (MRT)] were determined by classic techniques using Phoenix WinNonlin v7.0 software (Pharsight Corporation, Mountain View, CA). C_max_ (i.e., the maximum plasma concentration of moxifloxacin) and T_max_ (i.e., the time to reach maximum plasma drug concentration) were determined from the plasma drug concentration versus time profile. CL/F was determined as Dose/AUC, where CL = drug clearance and F = oral bioavailability of moxifloxacin.

Statistical differences among more than two mean values were determined by the Kruskal–Wallis one-way ANOVA test. Differences between any two mean values were evaluated statistically by unpaired Student's *t*-test or the Mann–Whitney U-test.

### Ethics statement

This study was carried out in accordance with the recommendation of the Guidelines for the Care and Use of Laboratory Animals (1996). All animal experiments were conducted with prior approval from the Texas Southern University Institutional Animal Care and Use Committee (IACUC).

## Data Availability

The pharmacokinetic data of this study is kept at Texas Southern University College of Pharmacy and Health Sciences.
